# Supplementary figures and images for: Optimisation of Sporosori Purification and Protein Extraction Techniques for the Biotrophic Protozoan Plant Pathogen Spongospora subterranea
Source: Molecules. 2020 Jul 8;25(14):3109. doi: 10.3390/molecules25143109 (PMC7397026; doi:10.3390/molecules25143109)

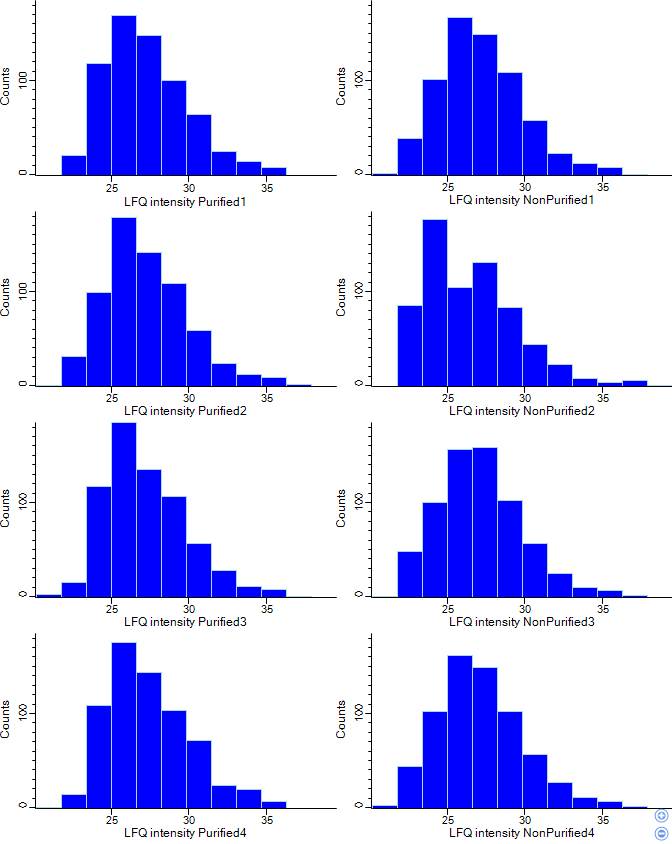

Supplement: Supplementary file 1 [file molecules-25-03109-s001.zip › S4- A histogram of intensity for each sample.png]
